# (*E*)-1-(5-Hy­droxy-2,2-dimethyl-2*H*-chromen-6-yl)-3-(3,4,5-trimeth­oxy­phen­yl)prop-2-en-1-one

**DOI:** 10.1107/S1600536811015236

**Published:** 2011-04-29

**Authors:** Guang-cheng Wang, Ying-hong Yang

**Affiliations:** a Department of Medicinal Chemistry, West China School of Pharmacy, Sichuan University, Chengdu 610041, People’s Republic of China; b Department of Pharmaceutical and Bioengineering, School of Chemical Engineering, Sichuan University, Chengdu 610065, People’s Republic of China

## Abstract

The title compound, C_23_H_24_O_6_, crystallizes with two independent mol­ecules (*A* and *B*) in the asymmetric unit. The dihedral angles between the benzopyran ring and the α,β-unsaturated ketone unit and between the α,β-unsaturated ketone group and the benzene ring are 9.4 (10) and 12.96 (13)°, respectively, in mol­ecule *A* and 1.40 (17) and 4.44 (17)°, respectively, in mol­ecule *B*. The two meth­oxy groups at the *meta* positions of the benzene ring are close to being coplanar with the ring [C—O—C—C = 6.2 (3) and −1.4 (3)° in mol­ecule *A* and −4.2 (4) and 3.7 (3)° in mol­ecule *B*], whereas the third meth­oxy group, at the *para* position, is (+)-anti­clinal with respect to the benzene ring [C—O—C—C = 81.7 (3)°] in mol­ecule *A* and is (−)-synclinal with respect to the benzene ring [C—O—C—C = −103.2 (3)°] in mol­ecule *B*. In both independent mol­ecules, the hy­droxy group is involved in an intra­molecular O—H⋯O hydrogen bond.

## Related literature

For the synthesis of related compounds, see: Krohn *et al.* (2002 ▶). For the biological activity of related compounds, see: Tran *et al.* (2009 ▶); Nerya *et al.* (2004 ▶). For related structures, see: Ranjith *et al.* (2010 ▶); Jasinski *et al.* (2009 ▶, 2010 ▶); Fun *et al.*(2010 ▶); Asiri *et al.*(2010 ▶).
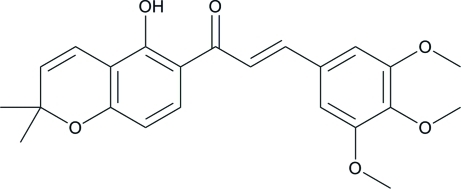


## Experimental

### 

#### Crystal data

C_23_H_24_O_6_
*M* *_r_* = 396.42
Triclinic, 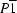

*a* = 9.9470 (9) Å
*b* = 13.9419 (13) Å
*c* = 16.1187 (11) Å
α = 72.681 (7)°
β = 89.487 (7)°
γ = 73.173 (8)°
*V* = 2035.3 (3) Å^3^
*Z* = 4
Mo *K*α radiation
μ = 0.09 mm^−1^
*T* = 293 K
0.22 × 0.15 × 0.15 mm

#### Data collection

Oxford Diffraction Xcalibur Eos diffractometer
Absorption correction: multi-scan (*CrysAlis PRO*; Oxford Diffraction, 2010 ▶) *T*_min_ = 0.952, *T*_max_ = 1.0
17082 measured reflections
8314 independent reflections
5047 reflections with *I* > 2σ(*I*)
*R*_int_ = 0.025

#### Refinement

*R*[*F* ^2^ > 2σ(*F* ^2^)] = 0.057
*wR*(*F* ^2^) = 0.148
*S* = 1.01
8314 reflections
541 parameters
H atoms treated by a mixture of independent and constrained refinement
Δρ_max_ = 0.45 e Å^−3^
Δρ_min_ = −0.24 e Å^−3^

### 

Data collection: *CrysAlis PRO* (Oxford Diffraction,2010 ▶); cell refinement: *CrysAlis PRO*; data reduction: *CrysAlis PRO*; program(s) used to solve structure: *SHELXS97* (Sheldrick, 2008 ▶); program(s) used to refine structure: *SHELXL97* (Sheldrick, 2008 ▶); molecular graphics: *OLEX2* (Dolomanov *et al.*, 2009 ▶); software used to prepare material for publication: *OLEX2*.

## Supplementary Material

Crystal structure: contains datablocks I, global. DOI: 10.1107/S1600536811015236/fl2342sup1.cif

Structure factors: contains datablocks I. DOI: 10.1107/S1600536811015236/fl2342Isup2.hkl

Supplementary material file. DOI: 10.1107/S1600536811015236/fl2342Isup3.cml

Additional supplementary materials:  crystallographic information; 3D view; checkCIF report

## Figures and Tables

**Table 1:** Hydrogen-bond geometry (Å, °)

*D*—H⋯*A*	*D*—H	H⋯*A*	*D*⋯*A*	*D*—H⋯*A*
O2—H2⋯O3	0.91 (3)	1.67 (3)	2.509 (2)	152 (3)
O8—H8⋯O9	0.98 (3)	1.64 (3)	2.536 (2)	149 (3)

**Fig. 1.**
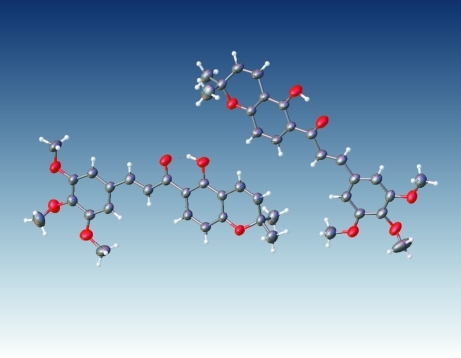
The molecular structure of the title compound, with displacement ellipsoids drawn at the 30% probability level.The intramolecular hydrogen bond is shown as a dashed line.

**Fig. 2.**
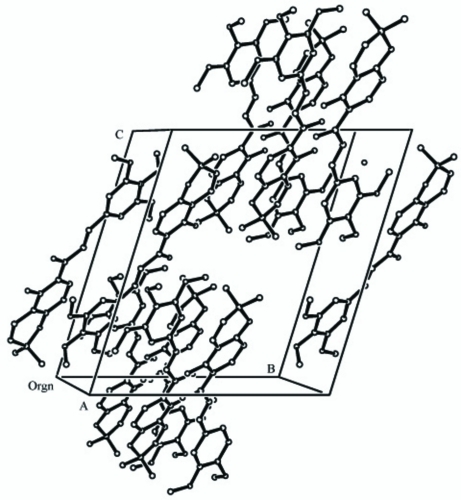
A crystal packing diagram of the title compound, viewed down the a axis.

## supplementary crystallographic information

### Comment

Chalcones (1,3-diaryl-2-propen-1-one) are natural or synthetic flavonoids displaying an impressive array of biological properties (Tran *et al.*, 2009; Nerya *et al.*,2004). The title compound, (I), is one of our synthetic chalcone derivatives which have shown anti-inflammation activity. The crystal structures of related compounds: (E)-1-[4-(Prop-2-yn-1-yloxy)phenyl]-3- (3,4,5-trimethoxyphenyl)prop-2-en-1-one (Ranjith *et al.*, 2010), (2E)-1-(2-Bromophenyl)-3- (3,4,5-trimethoxyphenyl)prop-2-en-1-one (Jasinski *et al.*, 2010), (E)-1-(2-Furyl)-3- (3,4,5-trimethoxyphenyl)prop-2-en-1-one (Fun *et al.*, 2010), (2E)-1-(4-fluorophenyl)-3- (3,4,5-trimethoxyphenyl)prop-2-en-1-one (Jasinski *et al.*, 2009), and (2E)-1-(2,5-Dimethyl-3- thienyl)-3-(2-methoxyphenyl)prop-2-en-1-one (Asiri *et al.*, 2010) have been reported. We report here the crystal structure of (I), a new chalcone.

(I) crystallizes with two independent molecules (A and B) in the asymmetric unit (Fig. 1). In one molecule, the dihedral angles between the benzopyran ring and the α,β-unsaturated ketone unit and between the α,β-unsaturated ketone group and the benzene ring are 9.42 (99) and 12.96 (13)°. The two methoxy groups at the meta positions of the benzene ring are close to being coplanar with the ring [C—O—C—C = 6.2 (3) and -1.4 (3)°], whereas the third methoxy group, at the para position, is (+)-anticlinal with respect to the benzene ring [C—O—C—C = 81.7 (3)°]. In the second molecule, the dihedral angles between the benzopyran ring and the α,β-unsaturated ketone unit and between the α,β-unsaturated ketone group and the benzene ring are 1.40 (17) and 4.44 (17)°. The two methoxy groups at the meta positions of the benzene ring are also close to being coplanar with the ring [C—O—C—C = -4.2 (4) and 3.7 (3)°], whereas the third methoxy group, at the para position, is (-)-synclinal with respect to the benzene ring [C—O—C—C = -103.2 (3)°]. In both independent molecules, the hydroxy group is involved in an intramolecular O—H···O hydrogen bond.

The crystal packing is shown in Fig. 2.

### Experimental

1-(5-hydroxy-2,2-dimethyl-2*H*-chromen-6-yl)ethanone(2.182 g,10 mmol), 3,4,5-trimethoxybenzaldehyde(1.962 g,10 mmol) in ethanol was added KOH (20% *w*/*v* aqueous solution) and the mixture was stirred at 273 K for 10 h. Then the crude product was recrystallized from ethanol to give (I). Single crystals suitable for X-ray structure determination were grown by slow evaporation of an ethyl ether solution of (II)) at room temperature.

### Refinement

H atoms were positioned geometrically (C—H = 0.93–0.96 Å) and refined using a riding model, with U*_iso_*(H) = 1.2 or 1.5U*_eq_*(C).

### Crystal data

**Table:**

C_23_H_24_O_6_	*Z* = 4
*M**_r_* = 396.42	*F*(000) = 840
Triclinic, *P*1	*D*_x_ = 1.294 Mg m^−^^3^
Hall symbol: -P 1	Mo *K*α radiation, λ = 0.7107 Å
*a* = 9.9470 (9) Å	Cell parameters from 4923 reflections
*b* = 13.9419 (13) Å	θ = 3.0–29.1°
*c* = 16.1187 (11) Å	µ = 0.09 mm^−^^1^
α = 72.681 (7)°	*T* = 293 K
β = 89.487 (7)°	Block, yellow
γ = 73.173 (8)°	0.22 × 0.15 × 0.15 mm
*V* = 2035.3 (3) Å^3^	

### Data collection

**Table:**

Oxford Diffraction Xcalibur Eos diffractometer	8314 independent reflections
Radiation source: fine-focus sealed tube	5047 reflections with *I* > 2σ(*I*)
graphite	*R*_int_ = 0.025
Detector resolution: 16.0874 pixels mm^-1^	θ_max_ = 26.4°, θ_min_ = 3.0°
ω scans	*h* = −12→12
Absorption correction: multi-scan (*CrysAlis PRO*; Oxford Diffraction, 2010)	*k* = −17→17
*T*_min_ = 0.952, *T*_max_ = 1.0	*l* = −20→20
17082 measured reflections	

### Refinement

**Table:**

Refinement on *F*^2^	Primary atom site location: structure-invariant direct methods
Least-squares matrix: full	Secondary atom site location: difference Fourier map
*R*[*F*^2^ > 2σ(*F*^2^)] = 0.057	Hydrogen site location: inferred from neighbouring sites
*wR*(*F*^2^) = 0.148	H atoms treated by a mixture of independent and constrained refinement
*S* = 1.01	*w* = 1/[σ^2^(*F*_o_^2^) + (0.0596*P*)^2^ + 0.3044*P*] where *P* = (*F*_o_^2^ + 2*F*_c_^2^)/3
8314 reflections	(Δ/σ)_max_ < 0.001
541 parameters	Δρ_max_ = 0.45 e Å^−^^3^
0 restraints	Δρ_min_ = −0.24 e Å^−^^3^

### Special details

**Table:**

Experimental. Empirical absorption correction using spherical harmonics, implemented in SCALE3 ABSPACK scaling algorithm.
Geometry. All e.s.d.'s (except the e.s.d. in the dihedral angle between two l.s. planes) are estimated using the full covariance matrix. The cell e.s.d.'s are taken into account individually in the estimation of e.s.d.'s in distances, angles and torsion angles; correlations between e.s.d.'s in cell parameters are only used when they are defined by crystal symmetry. An approximate (isotropic) treatment of cell e.s.d.'s is used for estimating e.s.d.'s involving l.s. planes.
Refinement. Refinement of *F*^2^ against ALL reflections. The weighted *R*-factor *wR* and goodness of fit *S* are based on *F*^2^, conventional *R*-factors *R* are based on *F*, with *F* set to zero for negative *F*^2^. The threshold expression of *F*^2^ > σ(*F*^2^) is used only for calculating *R*-factors(gt) *etc*. and is not relevant to the choice of reflections for refinement. *R*-factors based on *F*^2^ are statistically about twice as large as those based on *F*, and *R*- factors based on ALL data will be even larger.

### Fractional atomic coordinates and isotropic or equivalent isotropic displacement parameters (Å^2^)

**Table:**

	*x*	*y*	*z*	*U*_iso_*/*U*_eq_	
O1	0.95861 (16)	0.58077 (12)	0.27494 (11)	0.0589 (4)	
O2	0.9670 (2)	0.38689 (15)	0.07853 (14)	0.0811 (6)	
H2	0.905 (3)	0.386 (2)	0.038 (2)	0.109 (12)*	
O3	0.75683 (19)	0.44108 (14)	−0.02863 (12)	0.0787 (6)	
O4	0.27493 (17)	0.69344 (13)	−0.41625 (10)	0.0615 (4)	
O5	0.11331 (19)	0.87351 (13)	−0.39207 (11)	0.0719 (5)	
O6	0.11793 (16)	0.90604 (13)	−0.23882 (11)	0.0643 (5)	
O7	0.14385 (15)	0.83524 (12)	0.20823 (10)	0.0534 (4)	
O8	0.58354 (16)	0.66635 (13)	0.37529 (12)	0.0640 (5)	
H8	0.630 (3)	0.670 (2)	0.427 (2)	0.100 (10)*	
O9	0.62958 (17)	0.72834 (13)	0.50207 (11)	0.0678 (5)	
O10	0.67922 (18)	0.92060 (15)	0.87490 (12)	0.0742 (5)	
O11	0.45780 (18)	1.09188 (13)	0.84452 (11)	0.0668 (5)	
O12	0.25878 (18)	1.14008 (14)	0.71618 (12)	0.0754 (5)	
C1	1.0710 (2)	0.49482 (18)	0.33362 (15)	0.0525 (6)	
C2	1.1603 (2)	0.4292 (2)	0.28400 (17)	0.0645 (7)	
H2A	1.2543	0.3938	0.3040	0.077*	
C3	1.1095 (2)	0.4202 (2)	0.21254 (18)	0.0649 (7)	
H3	1.1655	0.3751	0.1848	0.078*	
C4	0.9655 (2)	0.48070 (17)	0.17680 (15)	0.0489 (5)	
C5	0.8962 (2)	0.56160 (17)	0.20991 (15)	0.0482 (5)	
C6	0.7635 (2)	0.62990 (19)	0.17363 (16)	0.0590 (6)	
H6	0.7182	0.6840	0.1960	0.071*	
C7	0.7014 (2)	0.61607 (18)	0.10495 (15)	0.0545 (6)	
H7	0.6133	0.6623	0.0807	0.065*	
C8	0.7642 (2)	0.53521 (17)	0.06891 (14)	0.0470 (5)	
C9	0.8980 (2)	0.46652 (17)	0.10828 (15)	0.0521 (6)	
C10	1.1532 (3)	0.5489 (2)	0.37303 (18)	0.0699 (7)	
H10A	1.0909	0.5933	0.4016	0.105*	
H10C	1.1943	0.5909	0.3278	0.105*	
H10B	1.2265	0.4968	0.4147	0.105*	
C11	1.0012 (3)	0.4300 (2)	0.40235 (19)	0.0855 (9)	
H11B	0.9375	0.4750	0.4298	0.128*	
H11C	1.0718	0.3771	0.4453	0.128*	
H11A	0.9499	0.3968	0.3756	0.128*	
C12	0.7010 (2)	0.52044 (18)	−0.00538 (15)	0.0535 (6)	
C13	0.5738 (2)	0.59855 (17)	−0.05740 (15)	0.0518 (6)	
H13	0.5242	0.6542	−0.0379	0.062*	
C14	0.5293 (2)	0.59073 (17)	−0.13112 (16)	0.0534 (6)	
H14	0.5809	0.5311	−0.1449	0.064*	
C15	0.4117 (2)	0.66076 (17)	−0.19463 (15)	0.0479 (5)	
C16	0.3968 (2)	0.63920 (17)	−0.27196 (15)	0.0503 (6)	
H16	0.4568	0.5784	−0.2801	0.060*	
C17	0.2941 (2)	0.70666 (17)	−0.33708 (14)	0.0474 (5)	
C18	0.2040 (2)	0.79777 (17)	−0.32491 (15)	0.0491 (5)	
C19	0.2145 (2)	0.81708 (17)	−0.24562 (15)	0.0474 (5)	
C20	0.3182 (2)	0.74967 (17)	−0.18115 (15)	0.0490 (5)	
H20	0.3255	0.7636	−0.1287	0.059*	
C21	0.3577 (3)	0.5985 (2)	−0.42989 (18)	0.0713 (7)	
H21B	0.3319	0.5979	−0.4869	0.107*	
H21A	0.3415	0.5394	−0.3867	0.107*	
H21C	0.4557	0.5939	−0.4252	0.107*	
C22	−0.0125 (3)	0.8552 (3)	−0.4090 (3)	0.1229 (15)	
H22C	−0.0726	0.9165	−0.4517	0.184*	
H22B	−0.0587	0.8406	−0.3562	0.184*	
H22A	0.0070	0.7961	−0.4309	0.184*	
C23	0.1245 (3)	0.9282 (2)	−0.15864 (17)	0.0744 (8)	
H23C	0.0571	0.9950	−0.1630	0.112*	
H23B	0.2175	0.9306	−0.1462	0.112*	
H23A	0.1034	0.8740	−0.1126	0.112*	
C24	0.1940 (2)	0.79416 (17)	0.13636 (14)	0.0474 (5)	
C25	0.3172 (2)	0.69796 (19)	0.16716 (15)	0.0543 (6)	
H25	0.3355	0.6491	0.1366	0.065*	
C26	0.4013 (2)	0.67979 (18)	0.23656 (15)	0.0510 (6)	
H26	0.4816	0.6216	0.2517	0.061*	
C27	0.3689 (2)	0.75053 (15)	0.28944 (13)	0.0404 (5)	
C28	0.2387 (2)	0.82697 (16)	0.27187 (13)	0.0402 (5)	
C29	0.1963 (2)	0.89413 (18)	0.32266 (15)	0.0514 (6)	
H29	0.1085	0.9449	0.3104	0.062*	
C30	0.2853 (2)	0.88433 (17)	0.39039 (14)	0.0480 (5)	
H30	0.2563	0.9290	0.4242	0.058*	
C31	0.4182 (2)	0.80980 (16)	0.41102 (13)	0.0411 (5)	
C32	0.4584 (2)	0.74255 (16)	0.35879 (14)	0.0425 (5)	
C33	0.0677 (2)	0.7725 (2)	0.10253 (17)	0.0644 (7)	
H33A	0.0915	0.7470	0.0535	0.097*	
H33C	−0.0097	0.8363	0.0848	0.097*	
H33B	0.0412	0.7204	0.1478	0.097*	
C34	0.2363 (3)	0.8798 (2)	0.06837 (16)	0.0701 (7)	
H34A	0.2732	0.8542	0.0212	0.105*	
H34C	0.3071	0.8988	0.0944	0.105*	
H34B	0.1553	0.9404	0.0466	0.105*	
C35	0.5126 (2)	0.79805 (17)	0.48459 (14)	0.0465 (5)	
C36	0.4706 (2)	0.86664 (17)	0.53995 (14)	0.0478 (5)	
H36	0.3858	0.9207	0.5248	0.057*	
C37	0.5486 (2)	0.85463 (17)	0.61035 (14)	0.0486 (5)	
H37	0.6340	0.8013	0.6219	0.058*	
C38	0.5175 (2)	0.91464 (16)	0.67238 (14)	0.0435 (5)	
C39	0.6137 (2)	0.88655 (17)	0.74345 (15)	0.0492 (5)	
H39	0.6944	0.8295	0.7514	0.059*	
C40	0.5909 (2)	0.94278 (18)	0.80282 (15)	0.0512 (6)	
C41	0.4718 (2)	1.02935 (18)	0.78994 (15)	0.0525 (6)	
C42	0.3737 (2)	1.05527 (18)	0.72055 (15)	0.0516 (6)	
C43	0.3958 (2)	0.99901 (17)	0.66142 (14)	0.0475 (5)	
H43	0.3297	1.0175	0.6145	0.057*	
C44	0.7986 (3)	0.8291 (2)	0.8931 (2)	0.0882 (10)	
H44B	0.8589	0.8370	0.8463	0.132*	
H44A	0.7677	0.7684	0.8987	0.132*	
H44C	0.8495	0.8201	0.9466	0.132*	
C45	0.3649 (3)	1.0768 (3)	0.9084 (2)	0.0914 (10)	
H45B	0.2702	1.1024	0.8816	0.137*	
H45C	0.3733	1.1144	0.9482	0.137*	
H45A	0.3869	1.0030	0.9395	0.137*	
C46	0.1505 (3)	1.1689 (2)	0.6485 (2)	0.0857 (9)	
H46B	0.1876	1.1887	0.5929	0.129*	
H46C	0.0748	1.2273	0.6539	0.129*	
H46A	0.1158	1.1102	0.6531	0.129*	

### Atomic displacement parameters (Å^2^)

**Table:**

	*U*^11^	*U*^22^	*U*^33^	*U*^12^	*U*^13^	*U*^23^
O1	0.0550 (9)	0.0592 (10)	0.0562 (10)	−0.0011 (8)	−0.0128 (8)	−0.0235 (8)
O2	0.0698 (12)	0.0721 (12)	0.0853 (15)	0.0268 (9)	−0.0305 (11)	−0.0468 (11)
O3	0.0773 (12)	0.0658 (12)	0.0781 (13)	0.0178 (9)	−0.0324 (10)	−0.0376 (10)
O4	0.0753 (11)	0.0568 (10)	0.0480 (10)	−0.0085 (9)	−0.0086 (8)	−0.0204 (8)
O5	0.0784 (12)	0.0620 (11)	0.0579 (11)	−0.0006 (9)	−0.0273 (9)	−0.0123 (9)
O6	0.0626 (10)	0.0598 (10)	0.0558 (11)	0.0107 (8)	−0.0178 (8)	−0.0241 (8)
O7	0.0457 (8)	0.0642 (10)	0.0484 (9)	−0.0021 (7)	−0.0095 (7)	−0.0283 (8)
O8	0.0503 (9)	0.0636 (11)	0.0725 (12)	0.0095 (8)	−0.0180 (9)	−0.0380 (10)
O9	0.0580 (10)	0.0694 (11)	0.0691 (12)	0.0064 (9)	−0.0233 (9)	−0.0351 (10)
O10	0.0670 (11)	0.0868 (13)	0.0653 (12)	0.0053 (10)	−0.0223 (9)	−0.0452 (10)
O11	0.0750 (11)	0.0725 (12)	0.0665 (12)	−0.0252 (10)	0.0109 (10)	−0.0384 (10)
O12	0.0628 (11)	0.0735 (12)	0.0735 (13)	0.0130 (9)	−0.0105 (10)	−0.0308 (10)
C1	0.0502 (13)	0.0545 (14)	0.0468 (14)	−0.0119 (11)	−0.0081 (11)	−0.0101 (11)
C2	0.0459 (13)	0.0719 (17)	0.0665 (18)	−0.0027 (12)	−0.0136 (12)	−0.0219 (14)
C3	0.0475 (13)	0.0687 (17)	0.0689 (18)	0.0069 (12)	−0.0110 (13)	−0.0307 (14)
C4	0.0393 (11)	0.0520 (13)	0.0470 (14)	−0.0011 (10)	−0.0049 (10)	−0.0149 (11)
C5	0.0443 (12)	0.0519 (13)	0.0449 (14)	−0.0088 (10)	−0.0012 (11)	−0.0151 (11)
C6	0.0480 (13)	0.0599 (15)	0.0621 (16)	0.0059 (11)	−0.0063 (12)	−0.0296 (13)
C7	0.0407 (12)	0.0547 (14)	0.0567 (15)	0.0063 (10)	−0.0077 (11)	−0.0203 (12)
C8	0.0422 (11)	0.0460 (13)	0.0437 (13)	−0.0016 (10)	−0.0049 (10)	−0.0118 (10)
C9	0.0476 (12)	0.0474 (13)	0.0509 (14)	0.0034 (10)	−0.0036 (11)	−0.0171 (11)
C10	0.0663 (16)	0.0778 (18)	0.0654 (18)	−0.0228 (14)	−0.0100 (14)	−0.0203 (15)
C11	0.093 (2)	0.094 (2)	0.069 (2)	−0.0460 (19)	0.0043 (17)	−0.0074 (17)
C12	0.0508 (13)	0.0474 (14)	0.0524 (15)	0.0002 (11)	−0.0072 (11)	−0.0150 (12)
C13	0.0476 (13)	0.0453 (13)	0.0532 (15)	−0.0013 (10)	−0.0076 (11)	−0.0136 (11)
C14	0.0537 (13)	0.0445 (13)	0.0525 (15)	−0.0017 (11)	−0.0104 (12)	−0.0134 (11)
C15	0.0467 (12)	0.0436 (12)	0.0474 (14)	−0.0095 (10)	−0.0071 (11)	−0.0088 (10)
C16	0.0539 (13)	0.0407 (12)	0.0521 (15)	−0.0075 (10)	−0.0060 (11)	−0.0143 (11)
C17	0.0549 (13)	0.0469 (13)	0.0390 (13)	−0.0167 (11)	−0.0036 (11)	−0.0097 (10)
C18	0.0509 (13)	0.0418 (12)	0.0447 (13)	−0.0076 (10)	−0.0121 (11)	−0.0046 (10)
C19	0.0441 (12)	0.0445 (13)	0.0480 (14)	−0.0065 (10)	−0.0066 (10)	−0.0127 (11)
C20	0.0490 (12)	0.0503 (13)	0.0421 (13)	−0.0081 (11)	−0.0064 (11)	−0.0126 (11)
C21	0.0831 (18)	0.0686 (18)	0.0656 (18)	−0.0154 (15)	−0.0047 (15)	−0.0327 (14)
C22	0.083 (2)	0.097 (3)	0.174 (4)	0.0011 (19)	−0.067 (2)	−0.043 (3)
C23	0.0760 (18)	0.0733 (18)	0.0635 (18)	0.0056 (14)	−0.0130 (15)	−0.0328 (15)
C24	0.0526 (13)	0.0534 (13)	0.0388 (13)	−0.0123 (11)	−0.0047 (10)	−0.0216 (11)
C25	0.0590 (14)	0.0584 (14)	0.0539 (15)	−0.0146 (12)	0.0008 (12)	−0.0325 (12)
C26	0.0492 (12)	0.0503 (13)	0.0543 (15)	−0.0053 (11)	−0.0029 (11)	−0.0265 (12)
C27	0.0415 (11)	0.0403 (12)	0.0395 (12)	−0.0102 (9)	0.0020 (10)	−0.0145 (10)
C28	0.0415 (11)	0.0439 (12)	0.0327 (11)	−0.0094 (9)	−0.0029 (9)	−0.0114 (9)
C29	0.0438 (12)	0.0546 (14)	0.0503 (14)	0.0021 (10)	−0.0065 (11)	−0.0244 (12)
C30	0.0509 (12)	0.0487 (13)	0.0416 (13)	−0.0022 (10)	−0.0052 (11)	−0.0222 (11)
C31	0.0435 (11)	0.0408 (11)	0.0360 (12)	−0.0084 (9)	−0.0038 (10)	−0.0114 (9)
C32	0.0393 (11)	0.0396 (12)	0.0443 (13)	−0.0040 (9)	−0.0026 (10)	−0.0140 (10)
C33	0.0624 (15)	0.0742 (18)	0.0652 (17)	−0.0236 (14)	−0.0076 (13)	−0.0306 (14)
C34	0.0864 (19)	0.0762 (18)	0.0512 (16)	−0.0344 (16)	−0.0058 (14)	−0.0146 (14)
C35	0.0496 (13)	0.0448 (12)	0.0431 (13)	−0.0099 (11)	−0.0065 (11)	−0.0147 (10)
C36	0.0503 (12)	0.0458 (13)	0.0444 (13)	−0.0086 (10)	−0.0075 (11)	−0.0152 (10)
C37	0.0529 (13)	0.0452 (13)	0.0463 (14)	−0.0110 (10)	−0.0074 (11)	−0.0152 (11)
C38	0.0477 (12)	0.0445 (12)	0.0397 (12)	−0.0139 (10)	−0.0036 (10)	−0.0146 (10)
C39	0.0455 (12)	0.0519 (13)	0.0500 (14)	−0.0058 (10)	−0.0065 (11)	−0.0238 (11)
C40	0.0493 (13)	0.0611 (15)	0.0455 (14)	−0.0110 (11)	−0.0082 (11)	−0.0248 (12)
C41	0.0538 (13)	0.0570 (14)	0.0523 (15)	−0.0127 (12)	0.0046 (12)	−0.0289 (12)
C42	0.0465 (12)	0.0526 (14)	0.0508 (15)	−0.0069 (11)	−0.0002 (11)	−0.0164 (11)
C43	0.0481 (12)	0.0519 (13)	0.0405 (13)	−0.0137 (11)	−0.0055 (10)	−0.0123 (11)
C44	0.0618 (16)	0.112 (2)	0.082 (2)	0.0119 (16)	−0.0299 (15)	−0.0532 (19)
C45	0.101 (2)	0.119 (3)	0.074 (2)	−0.041 (2)	0.031 (2)	−0.053 (2)
C46	0.0573 (16)	0.091 (2)	0.083 (2)	0.0113 (15)	−0.0130 (16)	−0.0219 (18)

### Geometric parameters (Å, °)

**Table:**

O1—C1	1.466 (3)	C19—C20	1.379 (3)
O1—C5	1.353 (3)	C20—H20	0.9300
O2—H2	0.91 (3)	C21—H21B	0.9600
O2—C9	1.343 (3)	C21—H21A	0.9600
O3—C12	1.249 (3)	C21—H21C	0.9600
O4—C17	1.365 (3)	C22—H22C	0.9600
O4—C21	1.421 (3)	C22—H22B	0.9600
O5—C18	1.373 (3)	C22—H22A	0.9600
O5—C22	1.392 (3)	C23—H23C	0.9600
O6—C19	1.365 (2)	C23—H23B	0.9600
O6—C23	1.422 (3)	C23—H23A	0.9600
O7—C24	1.462 (2)	C24—C25	1.486 (3)
O7—C28	1.356 (2)	C24—C33	1.516 (3)
O8—H8	0.98 (3)	C24—C34	1.518 (3)
O8—C32	1.349 (2)	C25—H25	0.9300
O9—C35	1.251 (2)	C25—C26	1.320 (3)
O10—C40	1.365 (3)	C26—H26	0.9300
O10—C44	1.425 (3)	C26—C27	1.454 (3)
O11—C41	1.392 (2)	C27—C28	1.386 (3)
O11—C45	1.387 (3)	C27—C32	1.393 (3)
O12—C42	1.371 (3)	C28—C29	1.395 (3)
O12—C46	1.422 (3)	C29—H29	0.9300
C1—C2	1.489 (3)	C29—C30	1.361 (3)
C1—C10	1.514 (3)	C30—H30	0.9300
C1—C11	1.510 (3)	C30—C31	1.395 (3)
C2—H2A	0.9300	C31—C32	1.412 (3)
C2—C3	1.316 (3)	C31—C35	1.459 (3)
C3—H3	0.9300	C33—H33A	0.9600
C3—C4	1.456 (3)	C33—H33C	0.9600
C4—C5	1.391 (3)	C33—H33B	0.9600
C4—C9	1.389 (3)	C34—H34A	0.9600
C5—C6	1.394 (3)	C34—H34C	0.9600
C6—H6	0.9300	C34—H34B	0.9600
C6—C7	1.361 (3)	C35—C36	1.468 (3)
C7—H7	0.9300	C36—H36	0.9300
C7—C8	1.405 (3)	C36—C37	1.320 (3)
C8—C9	1.417 (3)	C37—H37	0.9300
C8—C12	1.452 (3)	C37—C38	1.462 (3)
C10—H10A	0.9600	C38—C39	1.385 (3)
C10—H10C	0.9600	C38—C43	1.391 (3)
C10—H10B	0.9600	C39—H39	0.9300
C11—H11B	0.9600	C39—C40	1.386 (3)
C11—H11C	0.9600	C40—C41	1.390 (3)
C11—H11A	0.9600	C41—C42	1.382 (3)
C12—C13	1.472 (3)	C42—C43	1.383 (3)
C13—H13	0.9300	C43—H43	0.9300
C13—C14	1.317 (3)	C44—H44B	0.9600
C14—H14	0.9300	C44—H44A	0.9600
C14—C15	1.461 (3)	C44—H44C	0.9600
C15—C16	1.385 (3)	C45—H45B	0.9600
C15—C20	1.392 (3)	C45—H45C	0.9600
C16—H16	0.9300	C45—H45A	0.9600
C16—C17	1.380 (3)	C46—H46B	0.9600
C17—C18	1.393 (3)	C46—H46C	0.9600
C18—C19	1.393 (3)	C46—H46A	0.9600
			
O1—C1—C2	110.25 (19)	C16—C15—C14	118.3 (2)
O1—C1—C10	104.90 (18)	C16—C15—C20	119.4 (2)
O1—C1—C11	107.28 (19)	C16—C17—C18	119.6 (2)
O1—C5—C4	121.15 (19)	C17—O4—C21	117.74 (19)
O1—C5—C6	117.6 (2)	C17—C16—C15	121.0 (2)
O2—C9—C4	117.3 (2)	C17—C16—H16	119.5
O2—C9—C8	121.0 (2)	C18—O5—C22	116.4 (2)
O3—C12—C8	120.1 (2)	C19—O6—C23	116.84 (18)
O3—C12—C13	117.8 (2)	C19—C18—C17	119.5 (2)
O4—C17—C16	124.9 (2)	C19—C20—C15	120.0 (2)
O4—C17—C18	115.5 (2)	C19—C20—H20	120.0
O4—C21—H21B	109.5	C20—C15—C14	122.2 (2)
O4—C21—H21A	109.5	C20—C19—C18	120.4 (2)
O4—C21—H21C	109.5	H21B—C21—H21A	109.5
O5—C18—C17	121.2 (2)	H21B—C21—H21C	109.5
O5—C18—C19	119.0 (2)	H21A—C21—H21C	109.5
O5—C22—H22C	109.5	H22C—C22—H22B	109.5
O5—C22—H22B	109.5	H22C—C22—H22A	109.5
O5—C22—H22A	109.5	H22B—C22—H22A	109.5
O6—C19—C18	115.08 (19)	H23C—C23—H23B	109.5
O6—C19—C20	124.5 (2)	H23C—C23—H23A	109.5
O6—C23—H23C	109.5	H23B—C23—H23A	109.5
O6—C23—H23B	109.5	C24—C25—H25	119.0
O6—C23—H23A	109.5	C24—C33—H33A	109.5
O7—C24—C25	110.89 (17)	C24—C33—H33C	109.5
O7—C24—C33	104.36 (17)	C24—C33—H33B	109.5
O7—C24—C34	107.41 (17)	C24—C34—H34A	109.5
O7—C28—C27	121.41 (17)	C24—C34—H34C	109.5
O7—C28—C29	117.22 (18)	C24—C34—H34B	109.5
O8—C32—C27	117.30 (18)	C25—C24—C33	112.52 (19)
O8—C32—C31	121.38 (19)	C25—C24—C34	109.83 (19)
O9—C35—C31	120.40 (19)	C25—C26—H26	119.9
O9—C35—C36	119.13 (19)	C25—C26—C27	120.1 (2)
O10—C40—C39	124.7 (2)	C26—C25—C24	121.94 (19)
O10—C40—C41	115.59 (19)	C26—C25—H25	119.0
O10—C44—H44B	109.5	C27—C26—H26	119.9
O10—C44—H44A	109.5	C27—C28—C29	121.25 (19)
O10—C44—H44C	109.5	C27—C32—C31	121.30 (18)
O11—C45—H45B	109.5	C28—O7—C24	119.13 (16)
O11—C45—H45C	109.5	C28—C27—C26	117.70 (19)
O11—C45—H45A	109.5	C28—C27—C32	118.56 (18)
O12—C42—C41	114.28 (19)	C28—C29—H29	120.5
O12—C42—C43	125.0 (2)	C29—C30—H30	118.7
O12—C46—H46B	109.5	C29—C30—C31	122.55 (19)
O12—C46—H46C	109.5	C30—C29—C28	119.1 (2)
O12—C46—H46A	109.5	C30—C29—H29	120.5
C1—C2—H2A	119.2	C30—C31—C32	117.26 (19)
C1—C10—H10A	109.5	C30—C31—C35	122.54 (18)
C1—C10—H10C	109.5	C31—C30—H30	118.7
C1—C10—H10B	109.5	C31—C35—C36	120.46 (19)
C1—C11—H11B	109.5	C32—O8—H8	106.4 (17)
C1—C11—H11C	109.5	C32—C27—C26	123.67 (19)
C1—C11—H11A	109.5	C32—C31—C35	120.18 (19)
C2—C1—C10	112.1 (2)	C33—C24—C34	111.6 (2)
C2—C1—C11	110.8 (2)	H33A—C33—H33C	109.5
C2—C3—H3	120.0	H33A—C33—H33B	109.5
C2—C3—C4	120.1 (2)	H33C—C33—H33B	109.5
C3—C2—C1	121.6 (2)	H34A—C34—H34C	109.5
C3—C2—H2A	119.2	H34A—C34—H34B	109.5
C4—C3—H3	120.0	H34C—C34—H34B	109.5
C4—C5—C6	121.1 (2)	C35—C36—H36	118.7
C4—C9—C8	121.7 (2)	C36—C37—H37	115.9
C5—O1—C1	118.74 (17)	C36—C37—C38	128.2 (2)
C5—C4—C3	117.4 (2)	C37—C36—C35	122.6 (2)
C5—C6—H6	120.5	C37—C36—H36	118.7
C6—C7—H7	118.5	C38—C37—H37	115.9
C6—C7—C8	123.1 (2)	C38—C39—H39	119.7
C7—C6—C5	118.9 (2)	C38—C39—C40	120.6 (2)
C7—C6—H6	120.5	C38—C43—H43	120.1
C7—C8—C9	116.4 (2)	C39—C38—C37	118.4 (2)
C7—C8—C12	123.9 (2)	C39—C38—C43	119.57 (19)
C8—C7—H7	118.5	C39—C40—C41	119.7 (2)
C8—C12—C13	122.0 (2)	C40—O10—C44	117.36 (18)
C9—O2—H2	105 (2)	C40—C39—H39	119.7
C9—C4—C3	123.7 (2)	C40—C41—O11	119.1 (2)
C9—C4—C5	118.7 (2)	C41—C42—C43	120.7 (2)
C9—C8—C12	119.74 (19)	C42—O12—C46	117.54 (19)
H10A—C10—H10C	109.5	C42—C41—O11	121.2 (2)
H10A—C10—H10B	109.5	C42—C41—C40	119.64 (19)
H10C—C10—H10B	109.5	C42—C43—C38	119.7 (2)
C11—C1—C10	111.3 (2)	C42—C43—H43	120.1
H11B—C11—H11C	109.5	C43—C38—C37	122.0 (2)
H11B—C11—H11A	109.5	H44B—C44—H44A	109.5
H11C—C11—H11A	109.5	H44B—C44—H44C	109.5
C12—C13—H13	119.5	H44A—C44—H44C	109.5
C13—C14—H14	114.9	C45—O11—C41	115.46 (19)
C13—C14—C15	130.3 (2)	H45B—C45—H45C	109.5
C14—C13—C12	121.0 (2)	H45B—C45—H45A	109.5
C14—C13—H13	119.5	H45C—C45—H45A	109.5
C15—C14—H14	114.9	H46B—C46—H46C	109.5
C15—C16—H16	119.5	H46B—C46—H46A	109.5
C15—C20—H20	120.0	H46C—C46—H46A	109.5

### Hydrogen-bond geometry (Å, °)

**Table:**

*D*—H···*A*	*D*—H	H···*A*	*D*···*A*	*D*—H···*A*
O2—H2···O3	0.91 (3)	1.67 (3)	2.509 (2)	152 (3)
O8—H8···O9	0.98 (3)	1.64 (3)	2.536 (2)	149 (3)
